# Breast cancer and induced abortions in China

**DOI:** 10.1038/sj.bjc.6601853

**Published:** 2004-05-04

**Authors:** J Brind, V M Chinchilli

**Affiliations:** 1 Breast Cancer Prevention Institute, 9 Vassar Street, Poughkeepsie, NY 12603, USA; 2 Department of Health Evaluation Sciences, Pennsylvania State College of Medicine, Hershey, PA 17033, USA

**Sir**,

A recent study in this journal (Ye *et al*, 2002) has reported a slight but nonsignificant elevation in risk among Chinese women who had reported any induced abortions. On this basis, the authors conclude: ‘Abortions as they have been performed in China are not an important cause of breast cancer.’ This finding appears to be strengthened by the fact that the same odds ratio (OR=1.06) was obtained on their cohort of Shanghai textile workers either when a cohort analysis was performed, or when an age-matched case–control study was drawn from the cohort. The essentially null association was additionally reinforced by a similar finding in a previously published study on Shanghai women (Sanderson *et al*, 2001).

In China, it is well known that the government's ‘one child policy’ has led to a pattern in which induced abortion is very common, and almost always used after the first (usually, only) birth. Hence, women who are exposed to induced abortion would tend to be those who have their child(ren) at a younger age. The unexposed women, therefore, constitute a population that includes more women who are nulliparous or who had their child(ren) at a later age. Both of these characteristics are universally acknowledged risk factors for breast cancer, and were in fact observed by Ye *et al* in their cohort. The consequence of such population characteristics is confounding in the direction of underestimating the relative risk. That is, those women with induced abortion are at lower risk by virtue of the protection afforded by early childbirth, while those without induced abortion are at higher risk due to nulliparity or late childbirth. Consequently, adjustment for parity and age at first birth raises the relative risk estimate, as Ye *et al* observed. (The OR for the Ye *et al* cohort rises after adjustment for age and age at first birth from a raw value of 0.93 to 1.06) The opposite applies in a population such as in the US, where abortion is used predominantly to postpone first childbirth, rather than to limit family size.

Another important difference, however, between these Chinese study populations and those of most western industrialised countries, is the very high prevalence of induced abortion in China. In the study of Ye *et al*, the prevalence of induced abortion is 51%, and in the study of Sanderson *et al*, it is 66%. The validity of any observed association – null or otherwise – between a given exposure and a given disease outcome, rests upon, among other things, the unexposed population's serving as a typical, appropriate reference group. Once the prevalence of a given exposure rises to a level of predominance, it is prudent to ask whether indeed the unexposed comparison group has instead become a subgroup, which is unexposed for some reason that bears relevance to its risk profile for the disease in question. In such a case, statistical adjustment cannot remove all such confounding, since the calculation of the adjustment term will necessarily be underestimated. In the case of the Shanghai study population, the confounding by parity and age at first birth would not be fully corrected for, and the relative risk for induced abortion would remain underestimated.

Fortunately, the study design employed by Ye *et al* enables this hypothesis to be tested. In particular, the availability of a very large cohort of women (267 040) provided an ample supply of potential controls for the 702 eligible cases identified within the cohort. Indeed, Ye *et al* drew a control group that was closely age-matched by exact birth year. In both the cohort and case–control analyses, they found significant positive associations between age at first birth and breast cancer (data not shown) and nulliparity and breast cancer (relative risk (RR)=2.32, 95% CI: 1.45, 3.70 in the cohort analysis; case–control data not shown). As noted above, a nonsignificant association (RR=1.06) was found for induced abortion and breast cancer in both analyses. The hypothesis we propose in this letter, that the relative risk for induced abortion is underestimated in these analyses, can therefore easily be tested by drawing a new control group from the study cohort, wherein the controls are matched to cases not only by birth year, but also by parity and age at first birth. Such a case–control analysis, wherein controls are matched for these known confounders, would provide a more accurate estimate of the relative risk for induced abortion and breast cancer in this population.

In addition, there is another line of evidence, touched upon by Ye *et al*, which supports our presently proposed hypothesis. In particular, Ye *et al* suggest that their observed prevalence of induced abortion in their cohort (i.e. 51%) may be an underestimate, as it is substantially lower than the 66% reported by Sanderson *et al* (2001) in their earlier study. However, this discrepancy can easily be explained by differences in timing. Sanderson *et al*, although their paper was published earlier, actually studied an overlapping, but younger cohort (than that studied by Ye *et al*) of Shanghai women. Specifically, Ye *et al* studied women born between 1925 and 1958, whereas Sanderson *et al* studied women born between 1932 and 1973. Since the ‘one-child policy’ is of relatively recent vintage, dating back only to 1980, it is to be expected that the prevalence of induced abortion would be substantially higher in the Sanderson *et al* study population. Assuming, then, that the observed prevalence of induced abortion in both the Ye *et al* and Sanderson *et al* studies are accurate, we would also expect that confounding due to the high induced abortion prevalence would be greater in the Sanderson *et al* study. Consequently, the magnitude of underestimation of the relative risk should also be greater, that is, the observed relative risk should be lower. This is in fact the case. Sanderson *et al* (2001) reported an odds ratio of 0.9 for parous women, and 1.0 for all women (Sanderson *et al*, 2000) in the two published reports of their study.

Finally, the case made by Ye *et al* against there being a true positive association between induced abortion and breast cancer is not supported in the published record to the extent they suggest. They state: ‘No cohort studies (three are cited) or case–control studies nested within cohorts with ascertainment of abortion prior to development of breast cancer (two are cited) have shown associations of breast cancer with induced abortions.’ This claim is factually incorrect, since the prospective record-based case–control study of Howe *et al* (1989) – not cited at all by Ye *et al* – reported a statistically significant overall positive association (OR=1.9) between induced abortion and breast cancer. In fact, the overwhelming majority of published studies indicate a positive association between induced (but not spontaneous) abortion and breast cancer incidence (Brind *et al*, 1996). While it has been argued that some form of bias may be responsible for generating an apparent weak positive association (Lindefors-Harris *et al*, 1991), no credible evidence of such bias has been demonstrated. On the other hand, such confounding as we hypothesise in the present letter, can easily mask a true association, and we hope that Ye *et al* will take the opportunity to test for its presence in their analysis.
